# Highly Stretchable Micro/Nano Wrinkle Structures for Infrared Stealth Application

**DOI:** 10.1186/s11671-018-2783-z

**Published:** 2018-11-13

**Authors:** Jia Wang, Yijun Li, Jianli Cui, Hao Guo

**Affiliations:** 1 Department of Physics, School of science, North University of China, Taiyuan, 030051 Shanxi China; 2 Science and Technology on Electronic Test & Measurement Laboratory, North University of China, Taiyuan, 030051 Shanxi China

**Keywords:** Infrared stealth, Triangular wrinkle structures, Highly stretchable, Mechanical actuation

## Abstract

We demonstrate a novel infrared stealth structure consisting of SiO_2_/TiO_2_ film, which was manufactured as the highly stretchable triangular wrinkle structures. The triangular wrinkle structures have firstly been transferred to the flexible substrate from the surface of Si-substrate, which was manufactured by the MEMS technology. Then, the infrared reflective film have been manufactured to be the triangular wrinkle structures by depositing the materials (noble metal (Ag or Au) or multilayer oxide (SiO_2_/TiO_2_)) on the surface of flexible substrate. Due to the lower reflection effect of curved surface, the infrared reflectivity of these structures has been tuned down to 5%. And, compared to the flat surface, the reflection-to-diffuse ratios improved approximately one order of magnitude. These structures can adapt to the environment by changing the reflectivity of triangular wrinkle structures under stretching. Finally, an Au-modified infrared stealth structure has been fabricated as the array structures, which disappeared and then display by stretching the triangular wrinkle structures at room temperature. It features high reflection-to-diffuse ratios, stable repeatability, low-cost, and easy to manufacture. It may open opportunities for infrared camouflage for military security and surveillance field application.

## Background

Infrared stealth technology has been widely used in field of spacecraft components [1], camouflage platforms [2], protective clothing [3], container packaging [4], and so on [5–7]. Especially to the military security and military surveillance field, which can protect the aircraft from detection.

In recent year, many materials, which statically reflect radiation in the infrared region of the electromagnetic spectrum, have been studied [8–10]. Wei et al. [11] proposed a metamaterial-based infrared reflection method by modulating the photo-generated carrier doping. Kocabas et al. [12] demonstrate active surfaces structures that can be controlled to tune the reflection, transmission, and absorption of microwaves. However, this kind of novel material was confined by complex technology, ultra-low production, and high cost.

To optimize the adaptabilities of infrared stealth structures, many different adaptive infrared materials and structures have been designed and studied [13–15]. Valentine et al. [16] demonstrated a metamaterial-based infrared reflection method by spatiotemporally controlling the emissivity of metamaterial which is modulated with ultraviolet light. However, this structure has been actuated by the ultraviolet light, high temperatures, and large temperature gradients. Gorodetsky et al. [17] developed an adaptive infrared-reflecting structures based on the wrinkles structure with the feature of low working temperature, tuneable spectral range, fast response, and autonomous operation. However, this structure must be actuated by the high voltage about 3 kV, which is difficult to achieve in the common field, especially for aircraft.

In this study, a novel stretchable triangular wrinkle structure has been designed and manufactured to be the adaptive infrared stealth structure. The infrared reflectivity of this structure has been tuned to 5%, and a straightforward manufactured using the infrared reflecting materials was disappearance, and then display with the deformation of the triangular wrinkle structures under a simple mechanistically actuation at room temperature.

## Methods

The polydimethylsiloxane (PDMS) (10:1) membranes (Sylgard 184, Dow Corning) were prepared by spin coating on silicon wafers with a thickness of 500 μm by controlling the spinning speed, and cured immediately after spinning at less than 80 °C for 2 h [18].

The silver film and the alternating layers of titanium dioxide (TiO_2_) and silicon dioxide (SiO_2_) were deposited on the PDMS substrates by the electron-beam evaporation according to the standard micro-fabrication techniques.

The total reflectance, diffuse reflectance, and total transmittance of our structures were characterized with a Frontier transform infrared spectrometer (Perkin Elmer). The measurements were performed at an illumination angle of 12° over a wavelength range of 2 to 14 μm and referenced to a diffuse gold standard (Pike Technologies).

The topography of the triangular wrinkle structure were characterized by laser scanning microscope (Model: LEXT OLS4100; Co.: Olympus) and atomic force microscope (AFM) (Model: Multimode8; Co.: Bruker). The infrared pictures and videos were obtained with a thermal imager camera (FOTRIC 226S) for the temperature and an effective spectral range from wavelengths of 8 μm to 14 μm.

## Results and Discussion

### Infrared Stealth Mechanism

**Fig. 1:**
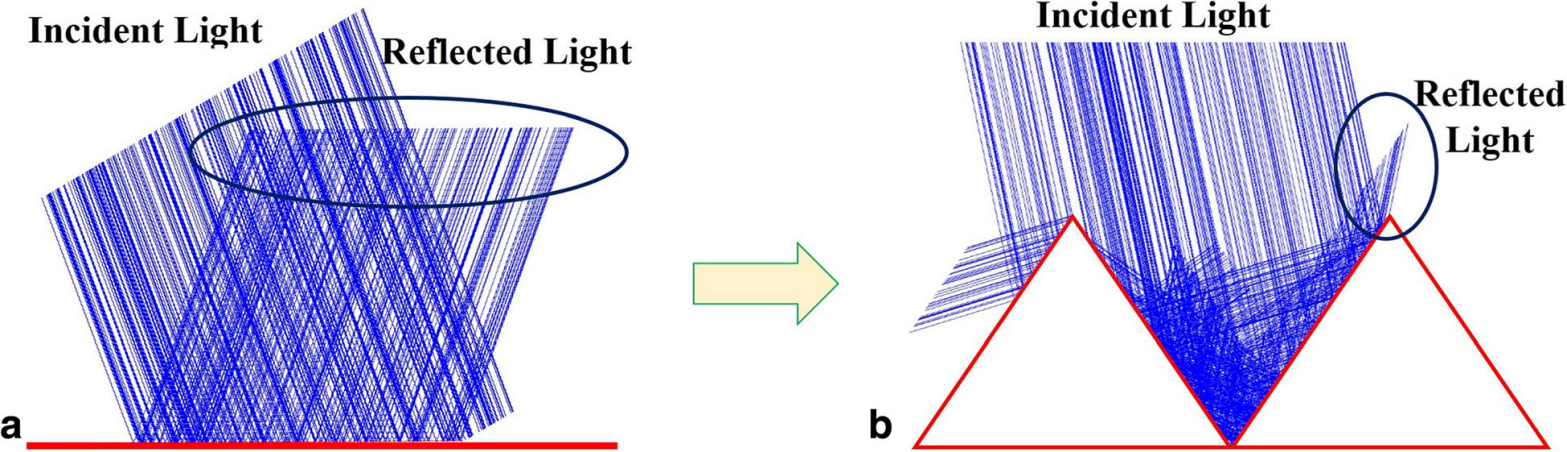
Model of infrared mechanism. **a** Light was incident on the flat film; **b** on the triangular structures

Schematics of the infrared reflecting structures were depicted in Fig. 1. We investigated the properties of infrared reflectance based on the triangular structure. The model of infrared stealth was simulated by the software of Zemax. As the light was incident on a flat structure, the most the incident light would reflected along a determined direction following the law of reflection, as shown in Fig. 1a. When the light was incident on the triangular structures, most of the light fall inside the triangular trap structures and only a litter light can reflect out of the triangular structures, as depicted in Fig. 1b. That is, as the infrared light was incident into the triangular structure, most of the infrared light would not be detected using the reflection mechanism of light. This triangular structure can be invisible for the infrared detection technique.

### Fabrication of Triangular Wrinkle Structure

**Fig. 2:**
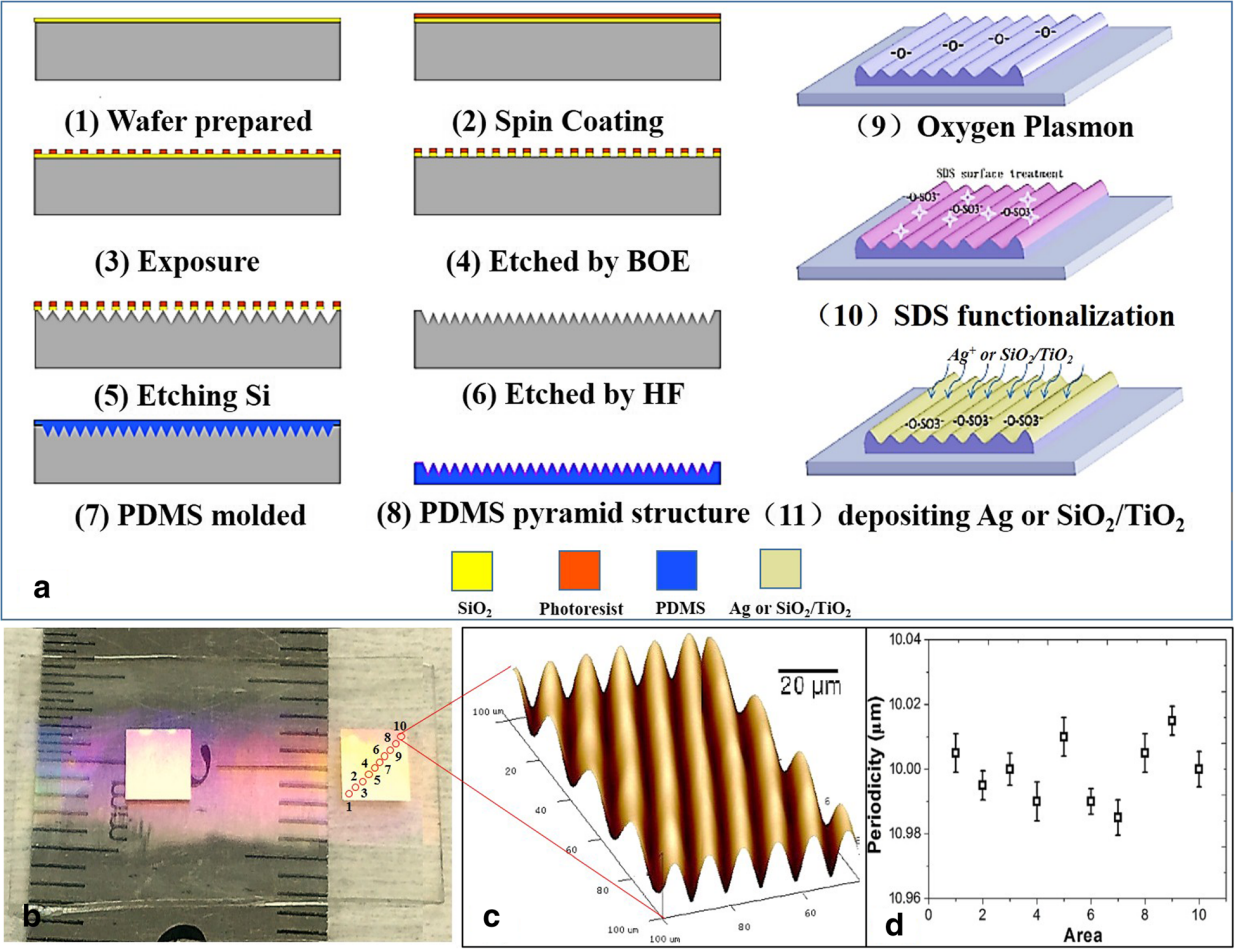
Fabrication process and morphology characterization of the triangular wrinkle structures on the polydimethylsiloxane (PDMS) substrates. **a** Fabrication process of the Ag (SiO_2_/TiO_2_)-embedded triangular wrinkle structures. **b** Optical image. **c** AFM image. **d** The uniformity of periodicity for the samples

As shown in Fig. 2, the triangular wrinkle structures were fabricated using the MEMS technology, which have been reported in our previous work [19]. First, a positive photoresist was spin-coated on the silica wafer at 3000 rad/min and baked at 105 °C for 90 s. Second, the wafer was exposed to a dose of 135 mJ/cm^2^ with a mask aligner and baked at 115 °C for 120 s to form strong crosslinks. After the wafer had gradually cooled down, the structure was immersed in a positive developer (40 s). Third, etch SiO_2_ using a buffered oxide etch and etch Si using 15 wt% TMAH + 17 vol% isopropyl alcohol (22 min). Fourth, a SiO_2_ layer was removed by the hydrofluoric acid. Then, a triangular-like structure was obtained, as shown in Fig. 2a [6]. Fifth, a PDMS mold was prepared by mixing the liquid PDMS elastomer and curing agent at a 10:1 ratio in volume, which was poured onto the Si mold and was thermally cured at 80 °C for 1.5 h to obtain the triangular structure on the surface of PDMS substrate to form the PDMS mold. Then, a SiO_x_ layer and hydrophilic groups (e.g., -OH) have been formed on the surface of the PDMS substrate under oxygen plasma treatment at 150 W for 15 s. The sample was then immersed in an SDS solution for 15 s to introduce -SO_3_^−^ groups at the surface of the triangular wrinkled PDMS structure. This process can introduce a condensation reaction of hydrophilic functionalities between the PDMS and noble metal (Ag, Au) and oxide materials (SiO_2_, TiO_2_), which have been reported in detail in our previous work [20–22]. Finally, metal or oxide triangular wrinkle structure were obtained by coating the metal or oxide on the surface of the PDMS mold using the electron-beam evaporation technology, which have been reported in detail in our previous work [20–22].

As shown in Fig. 2b–d, the periodicity of triangular wrinkle structures was uniform, and the periodicity was about (10 ± 0.1) μm on the whole surface of the sample tested by atomic force microscopy. And the size of sample was about 4 mm × 4 mm. Desired periodicity was achieved by tuning the size of mask structures and etch parameters, which can be calculated as introduced in our previous studies [19].

### Infrared Stealth Testing

In our work, the metal triangular wrinkle structures have been firstly fabricated to investigate the infrared reflection effect. Due to the high ductility, excellent bendability, and relatively low hardness and cost, metal Ag materials have been selected to manufacture the infrared reflective film. The Ag triangular wrinkle structures have been fabricated following the process of Fig. 2a.

**Fig. 3:**
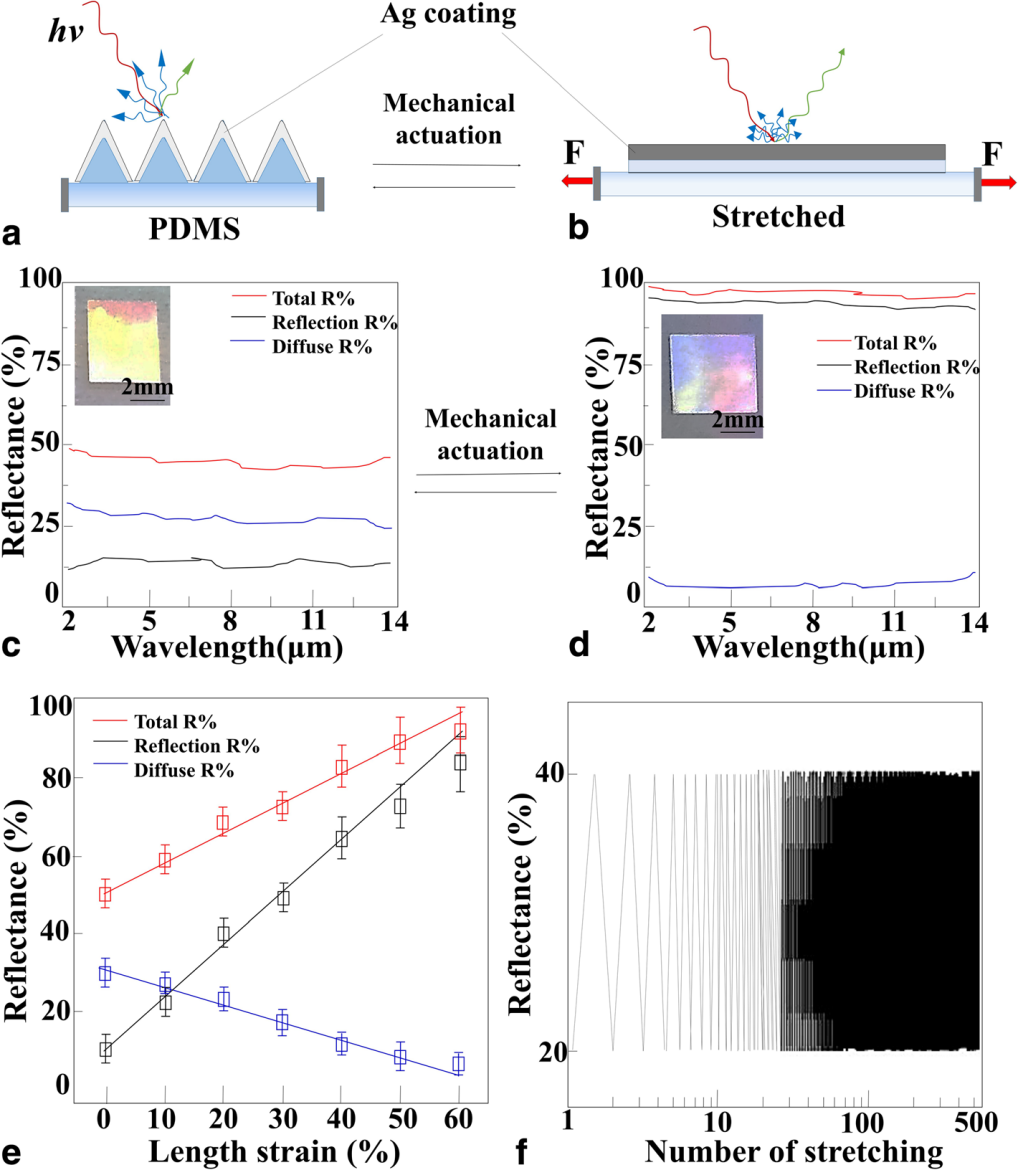
Mechanical modulation of the broadband reflectance. **a** The change in the surface morphology and the reflection of infrared light of the Ag triangular wrinkle structures before mechanical actuation. **b** After mechanical actuation. **c** The infrared reflectance spectra of the Ag triangular wrinkle structures before mechanical actuation. The total reflectance (red traces) are shown along with their reflection (black traces) and diffuse (blue traces) components. **d** After mechanical actuation. **e** Plots of the total, reflection, and diffuse peak reflectance of the Ag triangular wrinkle structures as functions of the applied length strain. **f** Stability test of the Ag triangular wrinkle structures with stretching/releasing over 500 cycles

Before mechanical actuation, when a beam of light is incident on the tip of the triangular infrared reflective structure (Fig. 3a), most of the infrared ray (red line) has been diffused by the tip (blue line) and only a little light can be reflected (green line) into the detector. While after mechanical actuation, the surface of triangular structure can be gradually stretched to be plane as shown in Fig. 3b. In the case, most of the incident light would reflect into the detector.

The corresponding infrared spectra have proved the above results in our experiment as shown in Fig. 3c. As unactuated triangular wrinkle structures, it featured a high average total reflectance of 46 ± 2%, a low average reflection of < 13%, and a moderate average diffuse light of 33 ± 2%. Hence, the total reflectance featured a weak average reflection of 13 ± 2% and a dominant average diffuse component of 33 ± 2%, in a ratio of ~ 0.4. After mechanically stretching the triangular wrinkle structures (as shown in Fig. 3d), the corresponding infrared spectra featured an increased average total reflectance of 97 ± 1%, a high average reflection of 89 ± 1%, and a low average total diffuse light of 8 ± 1%.

From the experiment results, the reflectivity increased from 13 to 89% with stretching the triangular Ag wrinkle structures. Also, the diffuse light has been reduced from 33 to 8%. The reason was that the triangular Ag wrinkle structures have been stretched to be Ag plane film. The incident light can reflect along a certain angle from the plane film following the law of reflection. Due to the high reflectivity of plane film, the total reflectance can be up to 100% in theory and the diffuse light was only a small amount. Considering the rough surface of Ag film, the reflection would be reduced (89%) and the diffused light would be increased (8%).

Meanwhile, compared to the wrinkle structure, the diffused light reduced from 33 to 8% from the plane film. The reason was that the roughness of the triangular Ag wrinkle structures was about ~ 1 μm depended by the height of wrinkle structures. But for the Ag plane film, the roughness was about ~ 20 nm or smaller, which was the roughness of Ag film. Hence, the diffuse light can be further reduced by optimizing the technological parameter of electron-beam evaporation.

In this case, the total reflectance featured a much larger average reflection of 89 ± 1% and a smaller average diffuse light component of 8 ± 1%, in a ratio of ~ 11. Hence, the reflection-to-diffuse ratios have been approximately increased an order of magnitude based on triangular wrinkle structures.

In general, the total reflectance of triangular wrinkle structures at broadband wavelength increased as a function of the strain (Fig. 3e). The reflectivity increased with the strain, but the diffuse reduced, as stretching the triangular wrinkle Ag film to be flat. The infrared reflection properties were fully reversible on repeated mechanical actuation contributing to the highly stretchable property of wrinkle structures. And only minor performance degradation has been observed after 500 cycles (Fig. 3f). Thus, mechanical actuation of our triangular wrinkle structures induced a change, which can be reversible and dynamically modulated of the broadband reflectance within the short- to long-wavelength infrared region.

In general, compare to the broadband infrared technology, the narrow infrared band has the higher signal-to-noise ratio and easier traceability for infrared target detection, discernment, and tracking application.

**Fig. 4:**
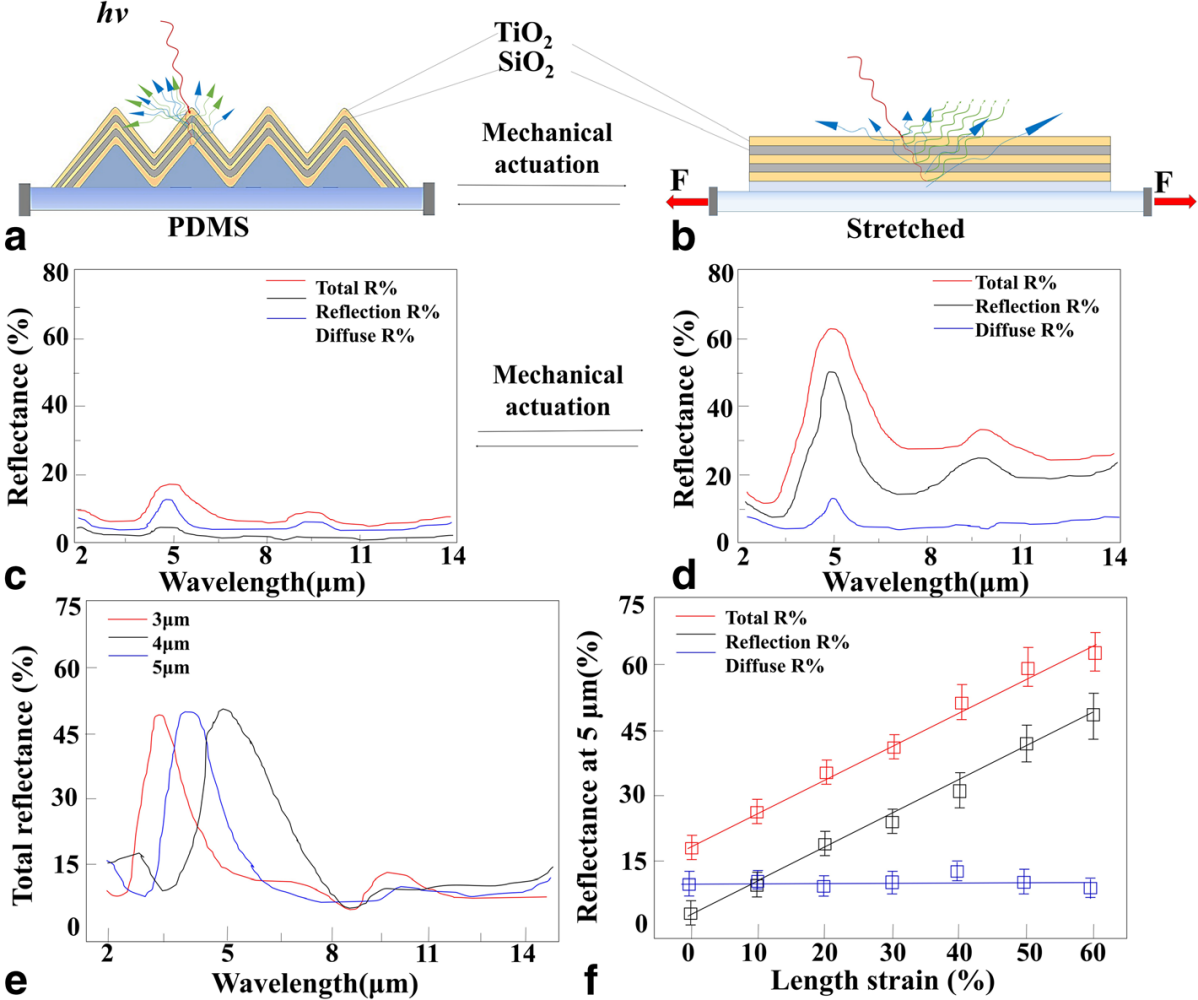
Mechanical modulation of the narrowband reflectance. **a** The change in the surface morphology and the reflection of infrared light of a TiO_2_/SiO_2_ Bragg stack-modified structures before mechanical actuation. **b** After mechanical actuation. **c** The infrared reflectance spectra of a TiO_2_/SiO_2_ Bragg stack-modified structures with a peak reflectance intensity at 5 μm before mechanical actuation. The total reflectance (red traces) are shown along with their reflection (black traces) and diffuse (blue traces) components. **d** After mechanical actuation. **e** The infrared spectra of three unactuated devices that have been designed to feature peak reflectance wavelengths of 3 μm (red trace), 4 μm (blue trace), and 5 μm (black trace). **f** Plots of the total, reflection, and diffuse peak reflectance of the TiO_2_/SiO_2_ Bragg stack-modified structures as functions of the applied length strain

Hence, to improve the precision of infrared target detection, discernment, and tracking technology, the narrowband infrared reflection effect of triangular wrinkle structures has been investigated as shown in Fig. 4.

To obtain narrowband infrared reflectance peak, alternating TiO_2_/SiO_2_/TiO_2_/SiO_2_/TiO_2_ layers with thicknesses of λ_peak_/(4 × *n*_*TiO2*_) and λ_peak_/(4 × *n*_*SiO2*_) have been designed. The structures were manufactured according to standard lithographic protocols as shown in Fig. 2a. The TiO_2_/SiO_2_ Bragg stacks with a peak reflectance intensity at 5 μm was produced with the SiO_2_ thickness was 0.933 μm and the TiO_2_ thickness was 0.543 μm by an Angstrom Engineering EvoVac system. The narrowband infrared reflection structures consist of two-layer SiO_2_ and three-layer TiO_2_. And the size of narrowband infrared reflection structures based on the triangular wrinkle structures was about 4 mm × 4 mm.

In our works, before mechanical actuation, the narrowband infrared reflection structures based on the triangular wrinkle structures, the infrared spectra featured the total reflectance intensities of 18 ± 2% at a wavelength of 5 μm, with a weak average reflection of 5 ± 2% and an average diffuse component of 13 ± 2%, in a ratio of ~ 0.38, as shown in Fig. 4c.

After mechanical actuation, the total reflectance intensities increased up to 63 ± 4% at a wavelength of 5 μm, with a much larger reflection component of 50 ± 3% and a nearly unchanged diffuse component of 13 ± 2% in a ratio of ~ 3.8 (as shown in Fig. 4d). This result was consistent with the Ag-modified reflective film based on the triangular wrinkle structures. The reflectivity increased from 5 to 63% caused by the reasons that the triangular TiO_2_/SiO_2_ multi-layers wrinkle structures have been stretched to be plane film. The incident infrared light can reflect along a certain angle from the plane film to improve the reflectivity.

Similarly, for narrowband infrared reflectivity structure based on the triangular wrinkle structures, the reflection-to-diffuse ratios reduced about one order of magnitude at a wavelength of 5 μm. Also, the same results can be proved at the wavelengths of 3, 4, and 5 μm (owing to the change of thickness of the TiO_2_ and SiO_2_), as shown in Fig. 4e.

In general, the total reflectance of triangular wrinkle structures at broadband wavelength increased as a function of the strain (Fig. 4f). The reflection increased with the strain, but the diffuse components remained relatively unaffected. The reason was that the roughness of the triangular wrinkle structures and the plane film was about the same size. The height of wrinkle structures was reduced from ~ 1 μm to ~ 200 nm with covering the TiO_2_ or SiO_2_ film. Because the corner between two triangular structures would cover more film than other area, which would reduce the height of TiO_2_/SiO_2_ multi-layers film wrinkle structures. The more the thickness of film increased, the more the height reduced. While for the TiO_2_/SiO_2_ plane film, the roughness was about ~ 50 nm caused by the worse quality for the oxide than the metal using the MEMS technology.

### Demonstration of Application Examples

As a proof-of-concept for infrared stealth of triangular wrinkle structures, we evaluated our infrared stealth structures to conceal themselves under infrared visualization.

**Fig. 5:**
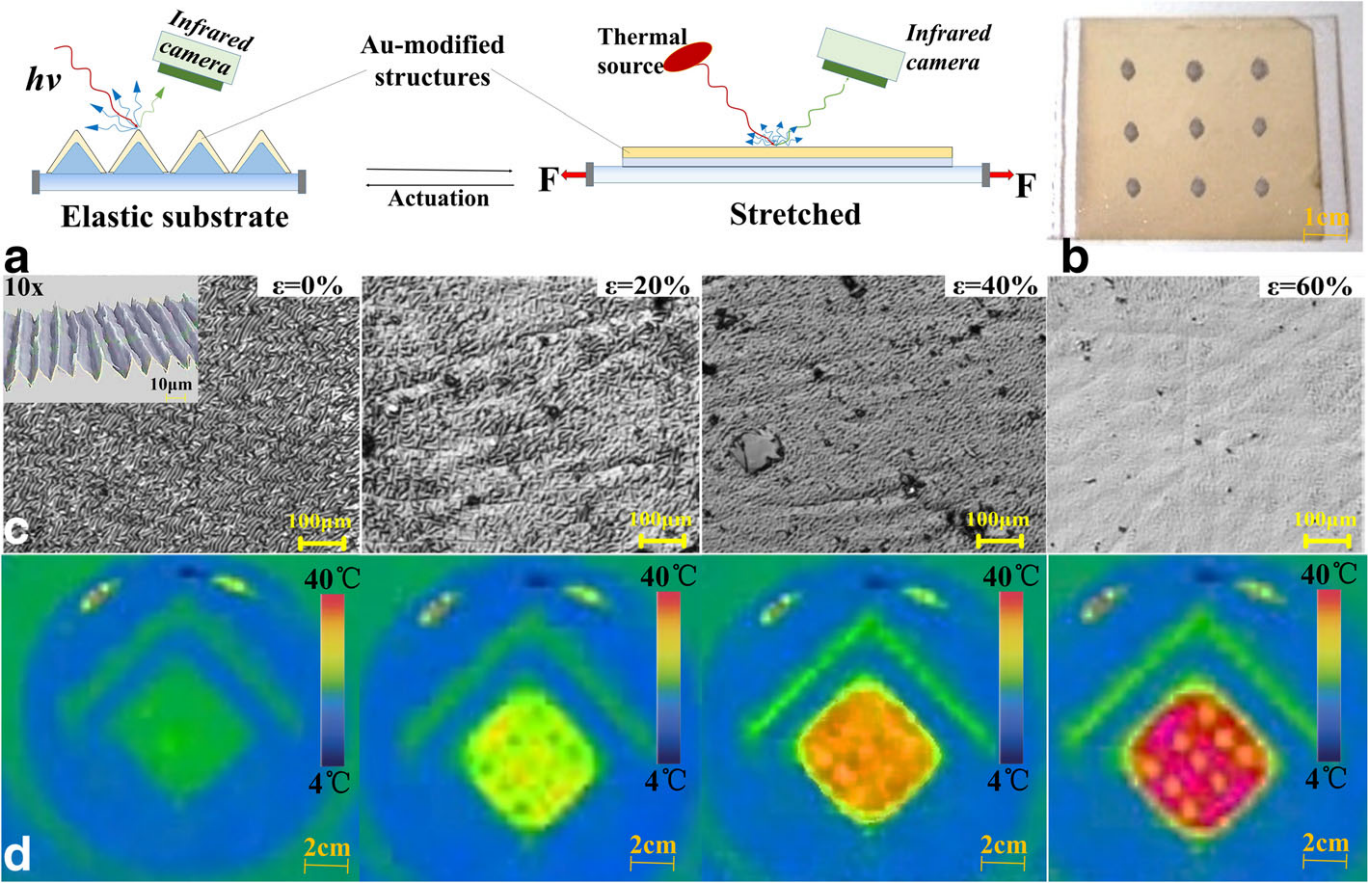
Reversible stealth of Au-modified structures in the infrared. **a** Schematics of an Au-modified structures under a constant thermal flux (left) before and (right) after mechanical actuation. **b** Optical image of Au-modified structures. **c** The microscopic morphological characteristics for triangular wrinkle structures with increasing the strain. **d** The corresponding infrared camera images of the same Au-modified structures with increasing the strain

We designed and manufactured an Au-modified infrared reflective film with three-by-three arrays structures. The size of the sample was 5 cm × 5 cm and imaged by a thermal infrared camera, as shown in Fig. 5a. The three-by-three arrays structures served as the label composed of PDMS-nanodiamond composite materials, which have high infrared transmission materials, as shown in Fig. 5b.

Before mechanical actuation, the triangular Au-modified wrinkle structures were clearly observed by the laser scanning confocal microscopy as shown in the inset in Fig. 5c (left). From the sectional view of inset, triangular structure can be seen clearly. And the corresponding infrared image can be shown in Fig. 5d (left). Without actuation, there was only an overall outline of Au-modified film structures. As the strain increased from 0 to 60%, the triangular wrinkle structure had been stretched to become flat and the height decreased to zero, which was observed by the laser scanning confocal microscopy. And the corresponding infrared image have shown that the Au-modified film structures gradually become red caused by the increased infrared reflectivity. And the three-by-three arrays structure was emerging to be a hole. Hence, the results prove the infrared stealth effect of the triangular wrinkle structures with the advantage of repeatability, stability, and fully reversibility.

## Conclusions

We have examined the infrared stealth properties of noble metal (Au and Ag) and metal oxide (TiO_2_/SiO_2_)-modified stretchable triangular wrinkle structures.

First, the infrared reflectivity of these structures has been tuned from 50 to 5% and the reflection-to-diffuse ratios dynamically modulated by approximately order of magnitude. Second, our presented structures feature capabilities for adaptive infrared camouflage technologies at the broadband and narrowband wavelength. Third, the structures have been straightforward integrated and featured stability to repeated cycling. Last, the structures enable new autonomous portable technologies under a simple mechanistically actuation at room temperature. Ultimately, the described structures may afford new possibilities for infrared camouflage applied in the field of military security and surveillance.

## Abbreviations

AFM: Atomic force microscope
PDMS: Polydimethylsiloxane
